# Integrating ankle and hip strategies for the stabilization of upright standing: An intermittent control model

**DOI:** 10.3389/fncom.2022.956932

**Published:** 2022-11-17

**Authors:** Pietro Morasso

**Affiliations:** Department of Robotics, Brain, and Cognitive Sciences, Center for Human Technologies, Istituto Italiano di Tecnologia, Genoa, Italy

**Keywords:** postural stabilization, double inverted pendulum, intermittent control, stiffness control, ankle strategy, hip strategy

## Abstract

Even in unperturbed upright standing of healthy young adults, body sway involves concurrent oscillations of ankle and hip joints, thus suggesting to using biomechanical models with at least two degrees of freedom, namely, a double inverted pendulum (DIP) framework. However, in a previous study, it was demonstrated that the observed coordinated ankle–hip patterns do not necessarily require the independent active control of the two joints but can be explained by a simpler hybrid control system, with a single active component (intermittent, delayed sensory feedback of the ongoing sway) applied to the ankle joint and a passive component (stiffness control) applied to the hip joint. In particular, the proposed active component was based on the internal representation of a virtual inverted pendulum (VIP) that links the ankle to the current position of the global center of mass (CoM). This hybrid control system, which can also be described as an ankle strategy, is consistent with the known kinematics of the DIP and, in particular, with the anti-phase correlation of the acceleration profiles of the two joints. The purpose of this study is to extend the hybrid control model in order to apply to both the ankle and hip strategy, clarifying as well the rationale of mixed strategies. The extension consists of applying the hybrid control scheme to both joints: a passive stiffness component and an active intermittent component, based on the same feedback signals derived from the common VIP but with independent parameter gains for the two joints. Thus, the hip gains are null in the pure ankle strategy, the ankle gains are null in the pure hip strategy, and both ankle and hip gains are specifically tuned in mixed strategies. The simulation of such an extended model shows that it can reproduce both strategies; moreover, the pure ankle strategy is more robust than the hip strategy, because the range of variation (RoV) of the intermittent control gains is larger in the former case than in the latter, and the pure ankle strategy is also more energy efficient. Generally, the simulations suggest that there is no advantage to employ mixed strategies, except in borderline situations in which the control gains are just outside the RoV that provides stable control for either pure strategy: in this case, a stable mixed strategy can emerge from the combination of two marginally unstable pure strategies.

## Introduction

Postural control strategies are known to be characterized by distinct muscle synergies, usually referred to as ankle strategy and hip strategy (Nashner and McCollum, 1985; Horak and Nashner, 1986; Kuo and Zajac, 1993). The ankle strategy involves delayed activation of the ankle muscles, followed by a distal to proximal activation of thigh and trunk muscles. The hip strategy consists mainly of the delayed activation of the trunk and thigh muscles, radiating in a proximal-to-distal fashion to other muscle groups. However, such muscle synergies are more evident in perturbed postural control, whereas in unperturbed upright standing the analysis of muscle activity carried out by Saffer et al. (2008) did not find any correlation between movements of the trunk and the activity of the muscles that exert direct control over it, whereas this correlation exists between ankle muscles and ankle oscillations.

Mechanically, the ankle strategy in unperturbed upright standing is usually described as a pure rotation of the body about the ankle joint with minimal movement about superior joints (Nashner and McCollum, 1985), allowing the body to act as a single-segment inverted pendulum (SIP model), controlled by ankle joint torque. A hip strategy involves the upper body rotating forward and downward, determining a backward rotation on the lower body (Runge et al., 1999). For healthy subjects standing on a stable, rigid surface, the ankle strategy is the default option and is based on the foot’s ability to exert, on the support surface, a torque that is (quasi) totally reflected on the standing body. The efficacy of the strategy may decrease if the support surface is not rigid or if it is narrow in comparison with the size of the foot: in the former case, part of the ankle torque is absorbed by the deformation of the surface; in the latter case, the small size of the support surface reduces the feasible range of motion of the center of pressure (CoP) of the ground reaction force, which is instrumental for allowing the ankle torque to dynamically counteract the toppling torque due to gravity, determined by the position of the center of mass (CoM) of the standing body.

In this context, the hip strategy is a second choice that is worth activating only when the ankle strategy is limited by the environmental conditions mentioned above, also because the two strategies operate according to different biomechanical mechanisms: the ankle strategy is mainly focused on the direct control of the CoP in relation with the CoM, whereas the hip strategy aims at the indirect control of the global CoM of the body by controlling the local CoM of the upper body. For this reason, the ankle strategy can also be named as a *CoP Strategy*, and the hip strategy can be seen as a *CoM Strategy* (Morasso, 2020).

During perturbed and unperturbed balance in standing, the most prevalent control strategy is the ankle strategy, which was found to be employed more than 90% of the time in balance (Blenkinsop et al., 2017). Moreover, the study clearly showed that little time is spent in a mixed strategy, representing less than 1% of time in unperturbed standing balance, although previous studies (Runge et al., 1999; Colobert et al., 2006) suggested the opposite.

Despite the general acceptance of the SIP model for describing the ankle strategy, recent studies clearly show that body sway in upright standing is not limited to rotations of the ankle joint: concurrent rotations around the hip joint are not negligible (Aramaki et al., 2001; Creath et al., 2005; Zhang et al., 2007), with comparable ranges of variation of the angular displacement, angular velocity, and angular acceleration of the ankle and hip joints. As a consequence, it has been suggested that the SIP model should be substituted by a multi-link paradigm, at least a double inverted pendulum (DIP) model, involving the coordinated control of ankle and hip joints. Ankle–hip joint coordination has been analyzed both in the time and frequency domains. In the former case, it was found that the acceleration profiles are strongly characterized by anti-phase patterns; the same holds, to a smaller degree, also for the velocity profiles, whereas the rotational profiles exhibit an overall mild in-phase correlation (Aramaki et al., 2001). Moreover, in the frequency domain, the rotational profiles of the two joints appear to be characterized by co-existing coordination patterns (in-phase and anti-phase, respectively) after a suitable frequency analysis (Creath et al., 2005; Zhang et al., 2007): the leg and trunk segments of the body move in-phase at low frequency (below 0.5 Hz) but they switch to anti-phase coordination at high frequency (above approximately 0.9 Hz).

Having accepted the fact that the SIP model misses out on part of the observable behavior, it remains an open question about the origin of the coordination between the two body segments of the DIP model in terms of fundamental mechanisms and control principles involved. One possibility (Sasagawa et al., 2009, 2014) is that the ankle–hip coordination during postural sway motion may be explained as an explicit attempt of the CNS to minimize the amplitude of the resultant angular acceleration of the CoM, by applying a multi-joint optimal control paradigm. In particular, it is hypothesized that the ankle torque and hip torque are explicitly modulated by the CNS in a temporally anti-phase manner to one another in each of the two joints in order to induce appropriate acceleration profiles. An alternative explanation (Morasso et al., 2019a) is that minimization of the CoM acceleration and the associated inter-joint coordination are not explicitly coded and controlled by the CNS but are the implicit biomechanical consequences of the dynamical interaction between the actively stabilized lower body and the passively stiffness-stabilized upper body. More specifically, the study quoted above was focused on the organization of the ankle strategy in the framework of a DIP paradigm, considering that sway movements involve oscillations of both joints determined by the interplay between opposing forces, namely, the destabilizing force of gravity, counteracted by the stabilizing effect of muscles that include two components: a passive component and an active component. The former one is related to the intrinsic mechanical properties of the muscular-tendinous tissues (Winters et al., 1988) with particular emphasis on stiffness; the latter active component consists in the activation of muscles due to sensory feedback of the ongoing sway, via proprioceptive, vestibular, and visual sensory signals. The main point, from the control point of view, is that the former component is virtually instantaneous, whereas the latter one is significantly delayed and delayed feedback in closed-loop control is a source of instability by itself, on top and on the side of the instability of the inverted pendulum posture.

For the ankle joint, whether in a SIP or DIP modeling framework, the crucial point is the value of the stiffness of the ankle muscles in relation to the rate of growth of the toppling torque due to gravity which thus identifies a *critical value of stiffness*. If the ankle stiffness were higher than such critical value, the stabilization of the SIP model would be assured in a purely passive manner without any need for an active feedback control mechanism: this is a *stiffness stabilization hypothesis* that was suggested by Winter et al. (1998). However, direct measurements of ankle stiffness (Loram and Lakie, 2002; Casadio et al., 2004) demonstrated that ankle stiffness is clearly under-critical, in a range of 60–80% of the critical value. A similar conclusion was reached by van Soest et al. (2003), based on a detailed neuromuscular model, and by (Fletcher et al., 2013), based on specific measurements of the stiffness of the Achilles tendon that emphasized the high compliance of the tendon. As a consequence, the CNS must supplement the passive compensation mechanism of gravity-driven instability with suitable active control strategies. A simple implementation of this mechanism is a conventional, linear, continuous-time feedback controller, based on proportional and derivative feedback (continuous *PD* control model) of the swaying body (Peterka, 2000; van der Kooij et al., 2001; Kiemel et al., 2002; Mergner et al., 2002; Masani, 2003). The cybernetic problem in this study is that such feedback information is delivered to the spinal and supra-spinal control centers through multiple sensory channels (proprioceptive, cerebellar, and visual) with a significant delay, well exceeding 0.2 s. In such conditions, the *PD* control parameters must be tuned carefully by considering two contrasting constraints: (1) the constraint of *static stability*, which dictates a minimum value of the *P* parameter as a function of the gravity toppling influence and (2) the constraint of *dynamic stability*, which imposes an upper bound for the *PD* parameters. Such contrasting constraints limit drastically the robustness of the continuous feedback *PD* control paradigm, motivating the formulation of a discontinuous version, namely, an intermittent feedback *PD* control mechanism (Bottaro et al., 2008; Asai et al., 2009, 2013), characterized by a simple switching mechanism defined in the phase plane of the body inverted pendulum ($q\,\mathrm{vs}.\dot{q}$, where *q* is the ankle rotation angle). The biomechanical rationale of this control model is that it can exploit the dynamic affordance provided by the saddle-like instability of the inverted pendulum: in saddle-like instability, it is possible to identify a stable and an unstable manifold in the phase plane defined above, which can be divided into four regions: two are fully unstable or unsafe regions and two metastable or safe regions. If the state vector of the SIP enters one of the unsafe regions it will monotonically diverge from equilibrium until fall; in the other case, the SIP would spontaneously approach equilibrium under the action of the stable manifold without any active control: this is the affordance provided by the saddle-type instability for a state-space intermittent feedback controller. Moreover, such dynamic affordance suggests a natural switching rule for exploiting it: as long as the current estimate of the state vector remains inside a safe region, the controller may turn off any control action (off-phase), letting the pendulum evolve at its natural pace, whereas it should switch on the feedback control action as soon as the state vector enters one of the unsafe regions (on-phase). The crucial point is that, during the on-phase, the purpose of the control action is not to attract the state vector toward the nominal equilibrium state but to the stable manifold. The bounded stability that can be achieved with this intermittent feedback approach is quite robust because it can work also with delayed information of the state vector, producing a limit cycle as an alternation of segments of hyperbolic orbits (off-phases) and spiral orbits (on-phases). In addition to the biomechanical motivation of the intermittent control paradigm, there is ample evidence suggesting the discontinuous nature of the feedback control action in upright standing: the analysis of posturographic patterns (Collins and De Luca, 1993; Morasso and Schieppati, 1999; Morasso and Sanguineti, 2002), EMG signals (Gatev et al., 1999; Runge et al., 1999; Cabrera and Milton, 2002; Asai et al., 2013), and the non-uniform character of sway path (Jacono et al., 2004). Remarkably, the intermittent control strategy can succeed to achieve bounded stability, driving the sway patterns toward a limit cycle, even if the dynamics of the on-phase is unstable when applied continuously, thus increasing in a substantial way the robustness of the intermittent control paradigm in comparison with the conventional continuous control paradigm (Asai et al., 2009, 2013).

In the previously quoted study (Morasso et al., 2019a), the hybrid stabilization control of the unperturbed standing posture of the SIP model, described above, was extended to a DIP paradigm with two working hypotheses: (1) the intermittent feedback control was applied to the ankle muscles with the specific indication that the phase plane used by the switching rule was not the one of the ankle joints ($q_{a\,n\,k\,l\,e}\,\text{vs}.{\dot{q}}_{a\,n\,k\,l\,e}$) but the plane of a virtual inverted pendulum (VIP) that links the ankle to the current position of the global CoM ($q_{C\,o\,M}\,\text{vs}.{\dot{q}}_{C\,o\,M}$)^1^; (2) the hip joint was supposed to have a stiffness greater than the critical value and thus stability of the upper body could be provided by the passive properties of the hip muscles, without any need for specific active control.^2^ As shown in that paper, the simulation of the DIP model controlled according to the two hypotheses above clearly shows that the experimental data about the inter-joint synergies of the DIP are fully accounted for by a simple hybrid control model: stiffness control plus intermittent feedback control of the ankle and pure stiffness control of the hip, without any explicit optimal coordination of the two joints. Moreover, the biological plausibility of this strategy is also consistent with the coherence analysis of muscle activity during quiet stance (Saffer et al., 2008) that shows a lack of correlation between the oscillations of the trunk and the activity of the muscles, which exert direct control over it.

In this study, the DIP coordination model is extended in order to include both ankle and hip strategies by allowing the intermittent control paradigm to be employed to the ankle, the hip, or both. Also in the proposed model, the controllers of the two joints are built on two components: a *passive component*, based on ankle stiffness (*K_a_* with the corresponding damping factor *B_a_*) and hip stiffness (*K_h_* with the corresponding damping factor *B_h_*), respectively; an *intermittent active component*, based on the delayed feedback of the positional error △*q*_δ_ and the corresponding speed error $△\,{\dot{q}}_{\delta }$ of the VIP, identified by the link that connects the global CoM to the ankle joint. In agreement with the previous study focused on the ankle strategy (Morasso et al., 2019a), the ankle stiffness is smaller and the hip stiffness is greater than the corresponding critical values. Both intermittent controllers operate on the delayed positional and speed errors of the VIP model defined above, with specific gain parameters: *P*_*a*_and*D*_*a*_ for the ankle intermittent controller; *P*_*h*_and*D*_*h*_ for the hip intermittent controller. In the framework of the proposed DIP coordination model, the pure ankle strategy is defined by the fact that the gain parameters of the hip controller (*P*_*h*_and*D*_*h*_) are both null, whereas in the pure hip strategy, the gain parameters of the ankle controller (*P*_*a*_and*D*_*a*_) are both null. Mixed strategies correspond to the fact that no one of the four gain parameters is null.

The main goal of this study was evaluating to which extent the simulation of the extended DIP model can reproduce the known behavior of both strategies, including in particular the anti-phase correlation of the acceleration profiles of the two joints. Moreover, it was interesting to assess the relative robustness of the two control strategies, in terms of the admissible range of variation of the control parameters capable to assure dynamics stability. An additional goal was to clarify the possible role of mixed strategies, considering the already quoted result (Blenkinsop et al., 2017) in unperturbed sway the mixed control choice is rather rare: this suggests that the mixed choice may emerge from environmental constraints such as the compliance of the standing surface or its limited size, in comparison with the foot size.

## Materials and methods

### The body model

**FIGURE 1:**
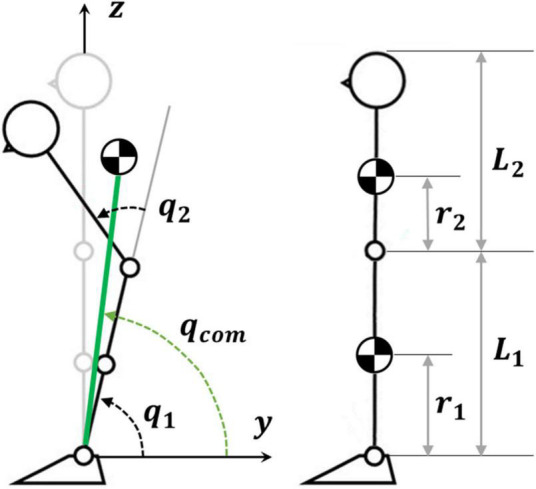
Biomechanical double inverted pendulum (DIP) model. The gray stick figure identifies the reference posture. The green line identifies the virtual inverted pendulum (VIP), i.e. the pendulum that links the ankle joint to the global CoM: *L*_*com*_ is the VIP length and *q*_*com*_ is the corresponding rotation angle.

As illustrated in Figure 1, the body is modeled as a double inverted pendulum with two joints (ankle and hip) and the corresponding links, related to the legs and the upper body or HAT (Head-Arm-Trunk). The model is characterized by the following anthropometric parameters, typical of a young adult male (Anthropology Research Project, 2000) with a weight of 84.14 kg and a height of 1.78 m:

Leg


$$m_{1}=\,\frac{1}{3}\,m_{t\,o\,t}=28.047\,k\,g$$

$$L_{1}=\,0.9\,m$$

$$r_{1}=\,0.64\cdot L_{1}=0.576\,m$$

I1=m1⁢r12=9.305⁢k⁢g⁢m2 (moment of inertia of the legs relative to a frame attached at the center of mass of the link, aligned with the *x*-axis).

HAT


$$m_{2}=\,\frac{2}{3}\,m_{t\,o\,t}=56.093\,k\,g$$

$$L_{2}=\,0.88\,m$$

$$r_{2}=\,0.36\cdot L_{2}=0.318\,m$$

I2=m2⁢r22=5.681⁢k⁢g⁢m2 (moment of inertia of the HAT relative to a frame attached at the center of mass of the link, aligned with the *x*-axis).

The two degrees of freedom of the model are the ankle rotation angle (*q_1_*) and the hip angle (*q_2_*). Moreover, for each time instant, the model is also characterized by a virtual link or VIP (Virtual Inverted Pendulum, green in Figure 1) that connects the ankle to the global CoM: *q*_*com*_ is the corresponding rotation angle. In the reference posture, *q*_1_ = *q*_*com*_ = 90*deg* and *q*_2_ = 0.

The dynamic equations of this double-inverted pendulum can be obtained by using the Lagrangian approach^3^ that yields the following non-linear ODE:


(1) $$\text{M}\,\left(q\right)\,\ddot{q}+\text{C}\,\left(q,\dot{q}\right)\,\dot{q}+\text{G}\,\left(q\right)=\tau$$

The motion of the model is specified by the joint rotation vector *q* = [*q*_1_*q*_2_] and the corresponding velocity and acceleration vectors ($\dot{q}$ and $\ddot{q}$). The motion is driven by the torque vector τ=[τ_1_τ_2_] that must match three dynamics elements:

• Inertial torque $\text{M}\,\left(q\right)\,\ddot{q}$
• Coriolis and centrifugal generalized torque $\text{C}\,\left(q,\dot{q}\right)\,\dot{q}$
• Gravity-dependent torque **G**(*q*)

The inertial matrix **M**(*q*)and the Coriolis matrix $\text{C}\,\left(q,\dot{q}\right)$ can be derived from the general expression of the kinetic energy function of the double pendulum:


$$\text{T}\,\left(q,\dot{q}\right)=\frac{1}{2}\,m_{1}\,\left({\frac{d\,{\hat{y}}_{1}}{d\,t}}^{2}+{\frac{d\,{\hat{z}}_{1}}{d\,t}}^{2}\right)+\frac{1}{2}\,I_{1}\,{\dot{q_{1}}}^{2}+\frac{1}{2}\,m_{2}\,\left({\frac{d\,{\hat{y}}_{2}}{d\,t}}^{2}+{\frac{d\,{\hat{z}}_{2}}{d\,t}}^{2}\right)+\frac{1}{2}\,I_{2}\,{\left(\dot{q_{1}}+\dot{q_{2}}\right)}^{2}=$$

(2) $$=\frac{1}{2}\,\left[\dot{q_{1}}\,\dot{q_{2}}\right]\,\left[\begin{array}{cc} A+2\,B\,c_{2} & D+B\,c_{2}\\ D+B\,c_{2} & D \end{array}\right]\,\left[\begin{array}{c} \dot{q_{1}}\\ \dot{q_{2}} \end{array}\right]$$

where $\left({\hat{y}}_{1},{\hat{z}}_{1}\right)$ are coordinates of the CoM of the leg, $\left({\hat{y}}_{2},\hat{z_{2}}\right)$ the coordinates of the CoM of the HAT; *s*_1_ = *sin*⁡*q*_1_; *s*_2_ = *sin*⁡*q*_2_; *s*_12_ = *sin*⁡(*q*_1_ + *q*_2_); *c*_12_ = *cos*⁡(*q*_1_ + *q*_2_); A=I1+I2+m1⁢r12+m2⁢(l12+r22); *B* = *m*_2_*l*_1_*r*_2_; D=I2+m2⁢r22. In particular, the following expressions of the two matrices can be derived:


(3) $$\text{M}\,\left(q\right)=\left[\begin{array}{cc} A+2\,B\,c_{2} & D+B\,c_{2}\\ D+B\,c_{2} & D \end{array}\right]$$

(4) $$\text{C}\,\left(q,\dot{q}\right)=\left[\begin{array}{cc} -B\,s_{2}\,{\dot{q}}_{2} & -B\,s_{2}\,\left({\dot{q}}_{1}+{\dot{q}}_{2}\right)\\ B\,s_{2}\,{\dot{q}}_{2} & 0 \end{array}\right]$$

Finally, the following expression of the gravity-dependent torque, which is the source of instability of the DIP model, can be derived from the potential energy component of the Lagrangian function:


(5) $$\text{G}\,\left(q\right)=-\frac{\partial U}{\partial q}=-g\,\left[\begin{array}{c} \left(m_{1}\,r_{1}+m_{2}\,l_{1}\right)\,c_{1}+m_{2}\,r_{2}\,c_{12}\\ m_{2}\,r_{2}\,c_{12} \end{array}\right]$$

This destabilizing torque is null in the reference posture and grows linearly with small displacements from the reference upright posture (δ*q*_1_δ*q*_2_).

### The control models

τ is the total control torque vector that has the main purpose to compensate the intrinsic instability of the upright posture due to the gravity-dependent torque (Equation 5) and stabilize the body around the reference posture. It includes three contributions, determined by different control mechanisms: a bias torque τ_*B*_, a stiffness torque τ*_S_*, and an intermittent feedback control torque τ_*I*_:


(6) $$\tau \;=\;\tau_{B}\;+\;\tau_{S}\;+\;\tau_{I}$$

A noise signal was added to the total control torque, with a comparable power and a limited frequency band: it was generated by sampling a normally distributed white noise source and filtering it with a 4th-order low-pass Butterworth filter (cutoff frequency 10 Hz). The two components of this torque vector are independent.

#### The feed-forward bias torque τ_B_

This torque compensates for the toppling torque due to gravity in the reference posture *q*_*ref*_, and it is applied to both joints according to a *feed-forward control strategy*, which is simply derived from Equation 5 by setting the two rotation angles to their reference values:


(7) $$\tau_{B}=-g\,\left[\begin{array}{c} \left(m_{1}r_{1}+m_{2}l_{1}\right)\cos q_{1\,r\,e\,f}+m_{2}r_{2}\cos\left(q_{1\,r\,e\,f}+q_{2\,r\,e\,f}\right))\\ m_{2}r_{2}\cos\left(q_{1\,r\,e\,f}+q_{2\,r\,e\,f}\right)) \end{array}\right]$$

For the simulations described in this study, the reference posture is fully vertical and thus the corresponding bias torque is null.

#### The stiffness torque τ_S_ and the stiffness control hypothesis

This torque vector expresses the viscous-elastic properties of muscles and tendons of the ankle and hip joints, respectively. In particular, the elastic component is assumed to be proportional to the distance of each joint angle from the corresponding reference value ($\delta \,q_{1}=q_{1}-q_{1}^{r\,e\,f}$ and $\delta \,q_{2}=q_{2}-q_{2}^{r\,e\,f}$):


(8) $$\tau_{S}=-\left[\begin{array}{c} K_{a}\,\delta \,q_{1}+B_{a}\,{\dot{q}}_{1}\\ K_{h}\,\delta \,q_{2}+B_{h}\,{\dot{q}}_{2} \end{array}\right]$$

*K_a_* represents the ankle stiffness (with the corresponding damping factor *B_a_*) and is a function of the level of coactivation of the antagonistic ankle muscles, whose balance codes the reference value of the ankle joint. The value of the ankle stiffness must be compared with the rate of growth of the toppling torque due to gravity acting on the ankle joint. Such parameter can be derived by linearizing Equation 5 around the reference posture ($q_{1}^{r\,e\,f}=90\,d\,e\,g$, $q_{2}^{r\,e\,f}=0$) yielding the following expression:


(9) $$\text{G}\,\left(\delta \,q\right)=g\,\left(m_{1}+m_{2}\right)\,\left[\begin{array}{c} L_{c\,o\,m}\\ r_{2}\,m_{2}/\left(m_{1}+m_{2}\right) \end{array}\right]\,\delta \,q_{1}+g\,m_{2}\,r_{2}\,\delta \,q_{2}$$

Here, *L*_*com*_ is defined as the distance from the ankle of the global CoM in the reference posture. Thus, we need to compare *K_a_* with *gm*_*tot*_*L*_*com*_ and it is immediate to conclude that a necessary condition for the asymptotic stability of the ankle joint, under a *stiffness control strategy*, is that *K*_*a*_≥*gm*_*tot*_*L*_*com*_. In other words, we can define *gm*_*tot*_*L*_*com*_ as the critical value of ankle stiffness: $K_{a}^{c\,r\,i\,t}$.

Direct methods of measuring the ankle stiffness (Loram and Lakie, 2002; Casadio et al., 2004) demonstrated that it is significantly smaller than the critical value, with a range of 60–80%. Thus, the stiffness control hypothesis of the ankle joint, suggested by Winter et al. (1998), is contradicted by empirical evidence.

*K_h_* is the hip stiffness (with the corresponding damping factor *B_h_*). In contrast with the ankle stiffness, there is no direct measurement of this parameter. However, there are reasons to hypothesize that the hip stiffness is greater than the critical value corresponding to the upper body (Morasso et al., 2019a): (1) the larger size of the hip muscles, in comparison with the ankle muscles and (2) the significantly smaller value of the critical hip stiffness in comparison with the ankle stiffness (175 Nm/rad vs. 654 Nm/rad). Thus, a value of $K_{h}=\mu_{h}\,K_{h}^{c\,r\,i\,t}$, which is twice the critical value (μ_*h*_ = 2), is a conservative choice, considering the size of the hip muscles; moreover, in the results section, there is a sensitivity analysis of the μ_*h*_ parameter. In the simulations considered in this study, the following values are used, together with the corresponding damping coefficients:


$$\left\{ \begin{array}{c} K_{a}=0.8\,K_{a}^{c\,r\,i\,t}=0.8\,g\,m_{t\,o\,t}\,L_{c\,o\,m}=523\,\mathrm{Nm}/\mathrm{rad}\\ K_{h}=2\,K_{h}^{c\,r\,i\,t}=2\,g\,m_{2}\,r_{2}=350\,\mathrm{Nm}/\mathrm{rad} \end{array}\right.$$

(10) $$\left\{ \begin{array}{c} B_{a}=30\,\mathrm{Nms}/\mathrm{rad}\\ B_{h}=44\,\mathrm{Nms}/\mathrm{rad} \end{array}\right.$$

#### The intermittent feedback control torque τ_I_

The feedback control law, as a complement of the passive stiffness stabilization mechanism of the gravity-dependent instability of the DIP model, is necessary because the intrinsic stiffness of one of the joints (the ankle) has an under-critical value. Three versions of the intermittent controlled were implemented:

1. Pure ankle strategy,
2. Pure hip strategy,
3. Mixed strategy (ankle and hip together).

In all the cases, the feedback control torques are computed as a function of the current delayed estimate of the state of the virtual inverted pendulum: *q*_*com*_(*t*−δ), ${\dot{q}}_{c\,o\,m}\,\left(t-\delta \right)$, where δ is the feedback delay. In the simulations, the following value of the feedback delay is used: δ=0.2*s*.

The rotation angle of the virtual inverted pendulum is reconstructed on-line from the two angles of the DIP system and the corresponding angular velocity is evaluated numerically:


$$\,\left\{ \begin{array}{c} x_{c\,o\,m}=\frac{m_{1}\,r_{1}+m_{2}\,L_{1}}{m_{1}+m_{2}}\,c_{1}+\frac{m_{2}\,r_{2}}{m_{1}+m_{2}}\,c_{12}\\ y_{c\,o\,m}=\frac{m_{1}\,r_{1}+m_{2}\,L_{1}}{m_{1}+m_{2}}\,s_{1}+\frac{m_{2}\,r_{2}}{m_{1}+m_{2}}\,s_{12} \end{array}\right.$$

(11) $$\Rightarrow q_{c\,o\,m}=t\,a\,n^{-1}\,\left(x_{c\,o\,m},y_{c\,o\,m}\right)$$

Thereafter, it is possible to compute the delayed angular error of the global CoM of the DIP model and the corresponding angular speed error:


(12) $$\left\{ \begin{array}{c} △\,q_{\delta }=\left(q_{c\,o\,m}^{r\,e\,f}-q_{c\,o\,m}\,\left(t-\delta \right)\right)\\ △\,{\dot{q}}_{\delta }=\left({\dot{q}}_{c\,o\,m}^{r\,e\,f}-{\dot{q}}_{c\,o\,m}\,\left(t-\delta \right)\right) \end{array}\right.$$

In the simulations, $q_{c\,o\,m}^{r\,e\,f}=90\,d\,e\,g$ and ${\dot{q}}_{c\,o\,m}^{r\,e\,f}=0$. As explained in previous studies (Bottaro et al., 2008; Asai et al., 2009, 2013), the rationale of delayed intermittent feedback control is to attenuate the risk of instability of traditional *PD* feedback controllers, due to the delay of the feedback signals, on top of the intrinsic instability due to gravity. The basic idea is to exploit the implicit “affordance” of saddle-like instability (typical of an inverted pendulum), namely the presence of a stable and unstable manifold in the phase plane, according to the following heuristics: to switch-off the feedback control action when the state vector is closer to the stable manifold than to the unstable one and to reactivate it in the opposite case. The robustness of this control paradigm is due to the fact that, even if the active control is unstable when permanently applied, the combination of actively controlled orbit segments with orbit segments driven by intrinsic dynamics may end up in bounded oscillatory patterns in a limited region of the phase plane. It is worth emphasizing that the target of active control, in the conventional continuous *PD* paradigm, is the upright unstable equilibrium configuration, whereas in the intermittent paradigm, it is the whole stable manifold, thus extending significantly the range of values (RoV) of the *PD* parameters that can support bounded stability (Asai et al., 2009).

#### Intermittent feedback control for the pure ankle strategy

The application of the feedback control law for the pure control strategy means that the active control is only applied to the ankle joint muscles, supporting the passive stabilizing effect of the ankle stiffness, whereas no active control is delivered to the hip, which can only count on muscle stiffness:


(13) $$\tau_{I}^{A-s\,t\,r\,a\,t}=\left[\begin{array}{c} A\,S\\ 0 \end{array}\right],\text{with}\,\left\{ \begin{array}{c} A\,S=P_{a}\cdot \Delta \,q_{\delta }+D_{a}\cdot \Delta \,{\dot{q}}_{\delta }\\ \text{if}\,\Delta \,q_{\delta }\cdot \left(\Delta \,{\dot{q}}_{\delta }-\Phi \,\Delta \,q_{\delta }\right)>0\\ A\,S=0\;\text{otherwise} \end{array}\right.$$

Here, [*P*_*a*_,*D*_*a*_] are the proportional and derivative parameters, respectively, of the *PD* intermittent controller for the ankle strategy, which is activated in the first and third quadrants of the phase plane of the virtual inverted pendulum, with an additional small slice determined by the parameter Φ. For this parameter, we used the value 0.4, in agreement with the analysis in Asai et al. (2009).

#### Intermittent feedback control for the pure hip strategy

The application of the feedback control law according to the pure hip strategy means that the active control is only applied to the hip joint muscles, further supporting the passive stabilizing effect of the hip stiffness, whereas no active control is delivered to the ankle, which can only count on muscle stiffness:


(14) $$\tau_{I}^{H-s\,t\,r\,a\,t}=\left[\begin{array}{c} 0\\ H\,S \end{array}\right],w\,i\,t\,h\,\left\{ \begin{array}{c} H\,S=P_{h}\cdot \Delta \,q_{\delta }+D_{h}\cdot \Delta \,{\dot{q}}_{\delta }\\ \text{if}\,\Delta \,q_{\delta }\cdot \left(\Delta \,{\dot{q}}_{\delta }-\Phi \,\Delta \,q_{\delta }\right)>0\\ H\,S=0\;\text{otherwise} \end{array}\right.$$

Here, [*P*_*h*_,*D*_*h*_] are the proportional and derivative parameters, respectively, of the *PD* intermittent controller for the hip strategy, which is activated in the first and third quadrants of the phase plane of the virtual inverted pendulum, in a similar way to the ankle strategy. Although the implementation of the two strategies suggests a symmetric control mechanism, the underlying biomechanics and the corresponding rationale are quite different. In the pure ankle strategy, the role of the active intermittent control is to shift the position of the CoP on the support base in such a way to anticipate the oscillations of the projection on the base of the global CoM. In contrast, the pure hip strategy does not allow active control of the CoP because the ankle joint does not transmit active torque to the ground. In this case, the active control of the upper body is to oscillate the local CoM of the upper body to keep the global CoM as close as possible to the vertical line.

#### Intermittent feedback control for a mixed ankle–hip strategy

The mixed ankle–hip strategy, in terms of intermittent feedback control, implies that both control actions are linked to the same activation rule, namely, the detection of a “dangerous” state of the VIP in the phase plane:


$$\tau_{I}^{M-s\,t\,r\,a\,t}=\left[\begin{array}{c} A\,S\\ H\,S \end{array}\right],\text{with}\,\left\{ \begin{array}{c} A\,S=P_{a}\,\Delta \,q_{\delta }+D_{a}\,\Delta \,{\dot{q}}_{\delta }\\ H\,S=P_{h}\,\Delta \,q_{\delta }+D_{h}\,\Delta \,{\dot{q}}_{\delta }\\ i\,f\,\Delta \,q_{\delta }\,\left(\Delta \,{\dot{q}}_{\delta }-\Phi \,\Delta \,q_{\delta }\right)>0 \end{array}\right.$$

(15)  ⁢A⁢S=H⁢S=0 otherwise

As will be clarified in the next section, this strategy, namely the need to add a part of hip strategy to an underlying weak ankle strategy, is motivated by the fact that the gains of the ankle controller are too small to stabilize the DIP.

The same effect can also be simulated by adding, in Equation 14, a soft-clipping function applied to *AS*. This function is characterized by a soft threshold value σ: when *AS* approaches or overcomes the threshold, the function yields a clipped version $\hat{A\,S}$ of the computed ankle torque to be transmitted to the ground:


(16) $$\left\{ \begin{array}{c} \begin{array}{c} \hat{A\,S}=A\,S-\frac{A\,S^{3}}{△}\,\text{for}-\sigma \leq A\,S\leq +\sigma \\ \hat{A\,S}=+\sigma -\frac{\sigma^{3}}{△}\,\text{for}\,A\,S>\sigma \end{array}\\ \hat{A\,S}=-\sigma +\frac{\sigma^{3}}{△}\,\text{for}\,A\,S<-\sigma \end{array}\right.$$

where △=3σ^2^.

The computational models described above were implemented in Matlab^©^ (MathWorks), and the simulations were carried out using the forward Euler method for integrating the following equation, with a time step of 0.001 s:


(17) $$\ddot{\text{q}}={\text{M}}^{-1}\,\left(-\text{C}\,\dot{\text{q}}-\text{G}+\tau_{B}+\tau_{S}+\tau_{I}+\text{noise}\right)$$

## Results

### Pure ankle and hip strategies

**FIGURE 2:**
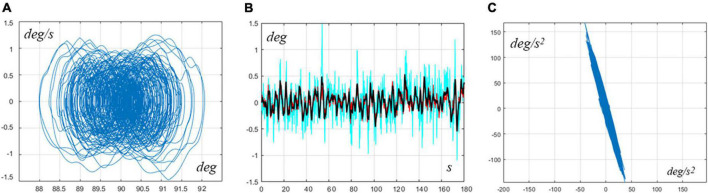
Typical pure ankle strategy. The simulation used the following intermittent control parameters: *P*_*a*_ = 875*Nm*/*rad*, *D*_*a*_ = 125*Nms*/*rad*, *P*_*h*_ = 0, and *D*_*h*_ = 0. **(A)** Typical sway orbits in the phase plane of the VIP underlying model ($q_{c\,o\,m}\,\mathrm{vs}.{\dot{q}}_{c\,o\,m}$). **(B)** Angular oscillations of the CoM angle (black), ankle angle (red), and hip angle (cian). **(C)** Angular acceleration of the ankle vs. angular acceleration of the hip, showing that the two variables are clearly in the antiphase. The simulation covered a time interval of 240 s, although only 180 s are plotted in panel **(B)** for convenience.

The first question we wished to answer was to evaluate the degree of robustness of the proposed hybrid postural controller to generate sway movements of the DIP model according to a pure ankle strategy (characterized by the fact that *P*_*h*_ = *D*_*h*_ = 0) as well as a pure hip strategy (characterized by the fact that *P*_*a*_ = *D*_*a*_ = 0). The ability of the hybrid control model to produce stable sway patterns according to the ankle strategy was already demonstrated in the previous study (Morasso et al., 2019a). Here, we evaluated the RoV of the ankle intermittent parameters that can produce bounded oscillatory patterns of the DIP model. The RoV of the two parameters is the following:

RoV for the pure Ankle Strategy: $P_{a}=408-2562\,\frac{N\,m}{r\,a\,d},D_{a}=0-1110\,\frac{N\,m\,s}{r\,a\,d}$. Thus, the RoV is rather large, indicating the solid robustness of the controller. In particular, Figure 2 is an example of typical sway patterns generated by the model in the middle of the parameter range ($P_{a}=875\,\frac{N\,m}{r\,a\,d},D_{a}=125\,\frac{N\,m\,s}{r\,a\,d}$): Panel A shows the typical sway orbits in the phase plane of the VIP underlying model ($q_{c\,o\,m}\,\text{vs}.{\dot{q}}_{c\,o\,m}$); panel B shows the angular oscillations of the CoM angle, ankle angle, and hip angle; panel C plots the angular acceleration of the ankle vs. the angular acceleration of the hip, showing that the two variables are clearly in antiphase.

**FIGURE 3:**
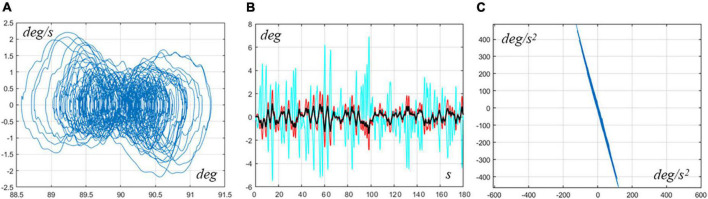
Typical pure hip strategy. The simulation used the following intermittent control parameters: *P*_*a*_ = 0, *D*_*a*_ = 0, *P*_*h*_ = 1120*Nm*/*rad*, and *D*_*h*_ = 305*Nms*/*rad*. **(A)** Typical sway orbits in the phase plane of the VIP underlying model ($q_{c\,o\,m}\,\mathrm{vs}.{\dot{q}}_{c\,o\,m}$). **(B)** Angular oscillations of the CoM angle (black), ankle angle (red), and hip angle (cian). **(C)** Angular acceleration of the ankle vs. angular acceleration of the hip, showing that the two variables are clearly in the antiphase. The simulation covered a time interval of 240 s, although only 180 s are plotted in panel **(B)** for convenience.

The simulation of the model for the pure hip strategy allowed us to identify the RoV of control parameters that generate bounded oscillations:

RoV for the pure Hip Strategy: $P_{h}=980-1260\,\frac{N\,m}{r\,a\,d},D_{h}=0-610\,\frac{N\,m\,s}{r\,a\,d}$. Thus, the pure hip strategy is feasible in the intermittent control framework but the RoV is smaller than for the ankle strategy. Figure 3 shows typical sway patterns generated with hip control parameters in mid-range: $P_{h}=1120\,\frac{N\,m}{r\,a\,d},D_{h}=305\,\frac{N\,m\,s}{r\,a\,d}$. The three panels of the figure display similar information as those of Figure 2. Remarkably, also for the hip strategy, the profiles of the angular acceleration of the ankle vs. the hip exhibit a counter-phase correlation.

The following table summarizes significant indicators extracted from the typical sway displayed in Figures 2, 3 for the two strategies.

In particular, the Table 1 shows that in the hip strategy the oscillation amplitude is largely greater for both joints and VIP angle. The amplitude of the control actions (related to stiffness and interactive control) is also higher, while compensating for a higher destabilizing torque due to gravity, while the amplitude of the noise was the same for both joints. The mean power delivered by the two active intermittent controllers was grossly higher in the hip strategy than in the ankle strategy: the power was estimated by multiplying the intermittent torque profile with the corresponding angular speed profile and then taking the average. Such rather low level of power depends also on the fact that intermittent control is energy-parsimonious by definition because the delivered torque is turned off for a significant fraction of time: in the two examples considered above, this percentage is 43.7% for the ankle strategy and 38.8% for the hip strategy.

**TABLE 1:** This table summarizes significant indicators extracted from the typical sway displayed in Figures 2, 3 for the ankle and hip strategies.

Pure ankle strategy—Typical DIP performance ($P_{a}=875\,\frac{N\,m}{r\,a\,d},D_{a}=125\,\frac{N\,m\,s}{r\,a\,d},P_{h}=0,D_{h}=0$)							
Standard dev *q*_*com*_	Standard dev *q_1_*	Standard dev *q_2_*	Standard dev τ_*grav*_	Standard dev τ_*stiff*_	Standard dev τ_*int*_	Standard dev τ_*noise*_	Mean ankle power
0.176 deg	0.152 deg	0.313 deg	2.464 Nm	1.360 Nm	2.347 Nm	2.844 Nm	0.0127 W

**Pure hip strategy—Typical DIP performance ($P_{a}=0,D_{a}=0,P_{h}=1120\,\frac{N\,m}{r\,a\,d},D_{h}=305\,\frac{N\,m\,s}{r\,a\,d}$)**							

**Standard dev *q*_*com*_**	**Standard dev** ***q_1_***	**Standard dev** ***q_2_***	**Standard dev τ_*grav*_**	**Standard dev τ_*stiff*_**	**Standard dev τ_*int*_**	**Standard dev τ_*noise*_**	**Mean** **ankle power**

0.466 deg	0.861 deg	1.933 deg	3.544 Nm	12.976 Nm	9.48 Nm	2.844 Nm	0.328 W

We also carried out a sensitivity analysis of the influence of the coefficient μ_*h*_ that characterizes the value of the hip stiffness in relation to the corresponding critical value. In most simulations reported in this paper, this value is 2 but given that no direct measurement is currently available we wished to verify how critical the choice of this value is.

In the case of the pure ankle stiffness strategy, it was found that increasing μ_*h*_ 100 times (from 2 to 200) has virtually no effect on the amplitude of the oscillations of the CoM: the standard deviation of *q*_*com*_ computed over 2 min interval remained virtually constant around a value of 0.176 deg and the slope of the ${\ddot{q}}_{1}\,\mathrm{vs}.{\ddot{q}}_{2}$ regression lines remained fixed around an average value of -3.68, emphasizing the robust anticorrelation of the acceleration profiles of the two joints. For decreasing values of μ_*h*_, it was found that, as expected, an instability was quickly reached (at μ_*h*_ = 1.2), with progressively increasing values of the of *q*_*com*_ standard deviation (0.24 deg, 0.28 deg, 0.40 deg, 0.43 deg, 0.55 deg) for decreasing values of μ_*h*_ (1.8, 1.6, 1.4, 1.3, 1.25).

In the case of the pure hip stiffness strategy, the range of values of μ_*h*_ around 2 that supported stable oscillations was much reduced: from 1.7 to 3. In this range, the standard deviation of *q*_*com*_ was about twice the standard deviation of the pure ankle stiffness case, whereas the slope of the ${\ddot{q}}_{1}\,v\,s.{\ddot{q}}_{2}$ regression lines was about the same.

### Mixed strategies

The first question we wished to answer was whether, in normal conditions, there was an advantage to use a mixed strategy over the pure ankle or hip strategies. If we combine the control parameters used for Figures 2, 3 in the same mixed strategy (*P*_*a*_ = 875, *D*_*a*_ = 125, *P*_*h*_ = 1120, *D*_*h*_ = 305), there is no advantage, also because the simulation shows that such mixed strategy is unstable. However, we found a critical advantage of using a suitable mixed strategy in borderline situations, i.e., situations in which the control parameters for the corresponding pure strategy are insufficient to achieve stability but the addition of a small contribution by the alternative strategy is enough to stabilize the oscillatory patterns.

**FIGURE 4:**
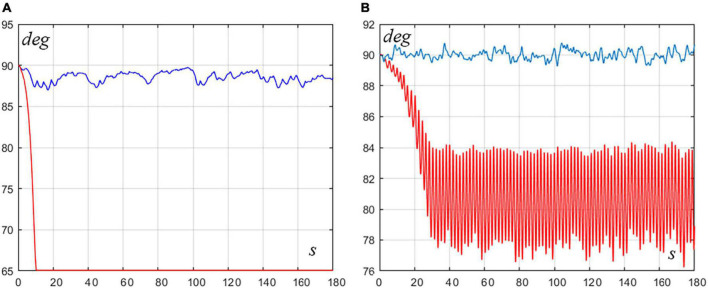
Examples of mixed strategies for borderline situations. Both panels display the time course of the VIP rotation angle (*q*_*com*_). **(A)** The red trace is related to an unstable pure ankle strategy (*P*_*a*_ = 375, *D*_*a*_ = 56, *P*_*h*_ = 0, *D*_*h*_ = 0) and the blue trace to a mixed strategy stabilized with a small contribution of hip strategy (*P*_*a*_ = 375, *D*_*a*_ = 55, *P*_*h*_ = 84, *D*_*h*_ = 7). **(B)** The red trace is related to an unstable pure hip strategy (*P*_*a*_ = 0, *D*_*a*_ = 0, *P*_*h*_ = 950, *D*_*h*_ = 105) and the blue trace to a mixed strategy stabilized with a small contribution of ankle strategy (*P*_*a*_ = 100, *D*_*a*_ = 80, *P*_*h*_ = 950, *D*_*h*_ = 105).

In particular, Figure 4 illustrates two examples. Panel A shows the time course of the rotation angle *q*_*com*_of the VIP model for a pure ankle strategy whose parameters (*P*_*a*_ = 375, *D*_*a*_ = 55, *P*_*h*_ = 0, *D*_*h*_ = 0) are outside the stability RoV (red trace): the sway is clearly unstable. In contrast, the blue trace of the same panel shows that a mixed strategy, with the same ankle strategy parameters including a rather small hip contribution (*P*_*a*_ = 375, *D*_*a*_ = 55, *P*_*h*_ = 84, *D*_*h*_ = 7) can achieve stability, although such hip strategy, alone, would be unstable. The robustness of this effect is supported by the fact that it persists if the parameters of the hip strategy are increased up to 100%. Panel B exemplifies a dual situation: the red trace is related to an unstable pure hip strategy (*P*_*a*_ = 0, *D*_*a*_ = 0, *P*_*h*_ = 950, *D*_*h*_ = 105) and the blue trace to a mixed strategy stabilized with a small contribution of ankle strategy (*P*_*a*_ = 100, *D*_*a*_ = 80, *P*_*h*_ = 950, *D*_*h*_ = 105). Again, both elements of the mixed strategy are unstable if applied separately although the combined mixed strategy is stable, and this effect persists if the parameters of the ankle strategy are increased by 100%. In summary, it appears that the motivation of adopting mixed strategies arises only in borderline situations, where the two combined strategies are individually outside the RoV of stability for pure control strategies.

**FIGURE 5:**
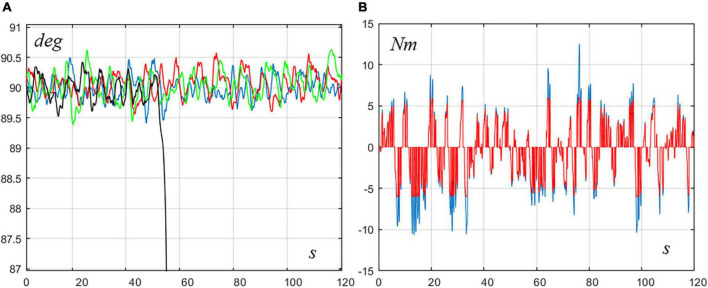
**(A)** Pure ankle strategy with various degrees of saturation of the intermittent control torque. The plotted curves refer to the VIP rotation angle *q*_*com*_. Blue: no saturation; Red: saturation = 9.5; Green: saturation = 9; Black: saturation = 8.5. The intermittent control parameters are: *P*_*a*_ = 875,*D*_*a*_ = 125,*P*_*h*_ = 0, and *D*_*h*_ = 0. **(B)** Intermittent control torque without saturation (blue trace) and with saturation (red trace, with a threshold of 8).

Finally, let us consider the effect of saturating the torque output of the intermittent controller of the ankle, under the pure ankle strategy, using a soft clipping function. Figure 5 shows the oscillation of the VIP angle *q*_*com*_ for different saturation thresholds. The underlying pure ankle strategy is characterized by the following parameters: *P*_*a*_ = 875*Nm*/*rad*, *D*_*a*_ = 125*Nms*/*rad*, and *P*_*h*_ = 0, *D*_*h*_ = 0. With these parameters, the standard deviation of intermittent torque is 2.43 Nm. The figure shows the oscillatory patterns for different values of saturation, in comparison with the unsaturated case. For values of the threshold equal to 9 Nm and above, the saturation has virtually no effect. However, a threshold of 8 Nm, which is larger than three times the standard deviation of intermittent torque, is sufficient to induce instability, although not instantaneously: it suggests that the threshold has only effect on the sparse spikes of the intermittent torque that exceeds three times the standard deviation. When this occurs, the fall is triggered. For more severe trimming thresholds, the fall is almost instantaneous. Both simulation paradigms clarify the limitation of the pure ankle strategy that is confirmed as the default choice for unperturbed upright standing but requires some degree of hip intervention when the reliability of foot grounding is decreased.

## Discussion

The hybrid control paradigm of upright unperturbed standing, based on a DIP model, recently proposed by Morasso et al. (2019a), was further confirmed by this study that was focused on the integration of ankle and hip strategies. First of all, it was demonstrated that the simulations of the extended DIP model can reproduce the known behavior of both strategies, including in particular the anti-phase correlation of the acceleration profiles of the two joints. In summary, it appears that the two strategies are indeed two instantiations of a common computational mechanism that, on one hand, exploits the intrinsic biomechanical properties of ankle and hip muscles and, on the other hand, activates an intermittent feedback compensation controller, applied to the ankle muscles, the hip muscles, or both. However, in all cases, the active control is driven by an internal estimate of the current state of the Virtual Inverted Pendulum. From this point of view, although the Single Inverted Pendulum model is an over-simplification that does not capture the observed ankle–hip patterns of sway movements, we agree with Gage et al. (2004) about the “kinematic and kinetic validity of the inverted pendulum in quiet standing” because it captures an essential part of the DIP dynamics, emphasizing the clinical importance of posturography (Visser et al., 2008) that is typically focused on the Single Inverted Pendulum model.

Moreover, the results show that the ankle strategy is much more robust than the hip strategy because the admissible range of variation of the control parameters for stability is much greater in the former case than in the latter. The ankle strategy also appears to be much more energy efficient than the hip strategy. As regard the interaction of the two strategies, the simulations show that for a stable ankle strategy little is gained by adding a concurrent activation of the hip strategy except when the gain parameters of the ankle strategy are near the lower bound of the admissible range or even a little bit beyond it: in this case, even a little amount of hip strategy can allow a faltering ankle strategy to recover stability. In our opinion, this may be interpreted as an environmental constraint to the achieved stability by the ankle strategy due to the compliance of the standing surface or its limited size, in comparison with the foot size. The same result, i.e., a reduced stability of the ankle strategy to be compensated by the recruitment of a suitable degree of hip active control, was also obtained by adding, in the simulation model, a soft saturation of the active ankle torque generated by the ankle intermittent controller. This clarifies the possible role of mixed strategies, considering the already quoted result (Blenkinsop et al., 2017) that in unperturbed sway the mixed control choice is rather rare.

At a more general level of analysis, it is important to note that the two fundamental elements of the proposed hybrid stabilization paradigm, namely intrinsic muscle stiffness and intermittent feedback control, are two basic building blocks that can be arranged and re-arranged by the brain differently according to different tasks and environmental conditions. The equilibrium point hypothesis (Feldman, 1966, 1986; Bizzi et al., 1992) clarified that any given posture is encoded and stabilized by antagonistic groups of muscles, via the selection of an appropriate coactivation level. The ankle joint is somehow an exception because of the high compliance of the Achilles tendon that is serially connected to the ankle muscles and thus strongly limits the chance of modulating the overall ankle stiffness by means of the coactivation of antagonistic muscles. However, although insufficient to fully compensate the gravity-dependent destabilizing torque, the ankle stiffness carries out a good part of the job, thus drastically reducing the necessary degree of attention by the CNS. There are balancing tasks where the stiffness component available to the brain is null by definition, for example, inverted stick balancing on the hand, and thus the direct responsibility as well as the required level of attention by the CNS is greatly increased. Nonetheless, the active stabilization of the two paradigms (upright standing vs. inverted stick balancing) can be explained with a similar intermittent feedback controller, despite the fact that the inverted pendulum models used by the controller are completely different (a Virtual Inverted Pendulum in one case and a physical stick in the other), as are different from the sensory feedback signals used by the brain for monitoring the state vector of the pendulum [mainly proprioceptive in one case (Peterka, 2002) and mainly visual in the other (Insperger and Milton, 2014)] and largely different is the required level of conscious and attentive control. In any case, the considerations above strongly support the view that the active part of the stabilization process is far from being a low-level reflex action: in contrast, it requires the integrative action of the CNS, including cognitive aspects as the internal representation of the body in relation with the environment (Morasso et al., 2015).

Another general issue about the ankle vs. hip strategies, on one side, and the relative role of CoP vs. CoM, on the other, is related to the “grounding” of the physical or virtual inverted pendulum. In the case of upright standing such “grounding” is provided by the feet: in spite of the common view that the feet are just a rigid base of support for the whole body, a recent study (Wright et al., 2012) clarified that the (naked) foot has a fundamental, double sensory-motor function: mechanically, it is compliant, sensitive to minute deformations, and with a friction coefficient that, all together, perfectly fit the need of a reliable transmission between the body and the ground of torques and forces; sensorially, the foot sole operates as a sensitive device of the distribution of contact forces, namely a kind of incorporated force platform that provides the brain an on-line estimation of the CoP. The ankle strategy for upright standing consists indeed in the modulation of the CoP position through the ankle torque, provided that the torque is firmly transmitted from the ankle muscles to the ground, to anticipate the sliding on the ground of the projection of the CoM. A totally equivalent strategy is adopted by skilled users when they succeed to balance an inverted stick on the hand or finger by shifting back and forth or side to side the hand, considering that the point of contact between the stick and the hand is the CoP in that paradigm (Morasso et al., 2019b). In other environmental conditions, like walking on a narrow beam or a tight rope the feasible range of movement of the CoP for medio-lateral sway is quite small and thus the equivalent of the ankle strategy (or CoP strategy) is not available to the brain. In that case, the equivalent of the hip strategy (or CoM strategy) is the only chance that indeed can be implemented in different ways, for example, using lateral shifts of a balance rod grasped with both hands (Morasso, 2020). The simulations performed in this study, with a hybrid, intermittent control paradigm, clarify the fact that there is little rationale for choosing a mixed strategy while the choice is dictated mainly by environmental constraints.

## Data availability statement

The raw data supporting the conclusions of this article will be made available by the authors, without undue reservation.

## Author contributions

The author conceived the study, wrote the manuscript, and developed the simulation software.

## Funding

This research was supported by internal funds of the RBCS (Robotics, Brain, and Cognitive Sciences) research unit of the Italian Institute of Technology, Genoa, Italy in the framework of the iCog initiative.

## Footnotes

1 Although the virtual inverted pendulum does not exist physically, its oscillations are directly perceivable by standing subjects through the CoP, namely by means of a combination of tactile and proprioceptive sensors of the feet.

2 The pure stiffness strategy for the hip joint is feasible because there is not the limitation of the Achilles tendon in the ankle joint; moreover, the critical stiffness value is strongly smaller for the hip than for the ankle case for purely biomechanical reasons, thus requiring a very small amount of co-contraction of the hip muscles for achieving a working level of hip stiffness.

3 Having defined the Lagrangian function $L\,\left(q,\dot{q}\right)$ as the kinetic energy (energy of motion) $T\,\left(q,\dot{q}\right)$ minus the potential energy (energy of position) *U*(*q*), the Lagrange equation of motion is $Q=\frac{d}{d\,t}\,\frac{\partial L}{\partial \bar{q}}-\frac{\partial L}{\partial q}$ where *q* is the vector of free coordinates and *Q* the corresponding vector of generalized forces.
